# The Predictive Role of Positive Mental Health for Attitudes Towards Suicide and Suicide Prevention: Is the Well-Being of Students of the Helping Professions a Worthwhile Goal for Suicide Prevention?

**DOI:** 10.1007/s10902-019-00163-1

**Published:** 2019-08-05

**Authors:** Patryk Stecz, Alena Slezáčková, Katarína Millová, Katarzyna Nowakowska-Domagała

**Affiliations:** 1 Department of Preventive and Addiction Psychology, Institute of Psychology, Faculty of Educational Sciences, University of Lodz, Smugowa 10/12, 91-433 Lodz, Poland; 2 Department of Psychology, Faculty of Arts, Masaryk University, Arna Novaka 1, 602 00 Brno, Czech Republic; 3 Institute of Psychology, Academy of Sciences of the Czech Republic, Veveří 97, 602 00 Brno, Czech Republic

**Keywords:** Positive mental health, Mental health problems, Psychological well-being, Attitudes towards suicide, Helping behavior, Hierarchical multiple regression, Potential gatekeepers

## Abstract

This study evaluates the potential value of eudaimonic well-being in assessing pro-preventive orientation towards suicide and recognizing suicide as a solution. The aim was to integrate positive and negative conceptualizations of mental health for predicting attitudes towards suicide, and towards suicide prevention, among students of the helping professions. The study participants (166 women and 73 men, mean age 22.84 ± 5.15) answered a set of questionnaires, including a Questionnaire on Attitudes Towards Suicide, Goldberg Health Questionnaire (GHQ-28), Psychological Well-Being Scale (PWB-42) and Centrality of Religiosity Scale. Multiple regression analysis showed that environmental mastery, purpose in life and positive relationships, controlled for religiousness and psychological problems related to general mental health, predicted the variability of attitudes towards suicide and pro-preventive orientation. Sociodemographic variables were not related to attitudes towards suicide. Our findings suggest that positive mental health, represented jointly by low mental health problems and eudaimonic components of happiness, plays a role in predicting pro-preventive attitudes. Therefore, improving positive mental health among students in the helping professions, these being the future gatekeepers, could be considered an auxiliary strategy for suicide prevention.

**Electronic supplementary material:**

The online version of this article (10.1007/s10902-019-00163-1) contains supplementary material, which is available to authorized users.

## Introduction

The understanding of suicidal behavior has been recently influenced by concepts originating from positive psychology, which served to synthesize theories organized around the topic of prevention (Barnes 2017; Hirsch et al. 2018). Positive suicidology aims at identifying protective factors for suicidal behavior and examining useful constructs from positive psychology to increase the robustness of suicide prevention (Wingate et al. 2006). Identifying those at risk is an important goal and suicide prevention is a challenging task faced by the entire community, including groups of gatekeepers. Together, their attitudes towards suicide (ATS), suicide prevention (ATSP) and mental health constitute a personal background that can be brought to the intervention, and as such, merit more research interest within the area of positive suicidology.

Professional gatekeepers comprise healthcare providers (designated gatekeepers) and specialists from outside the health sector (emergent groups such as counselors, clergy, teachers) who are likely to interact with people at higher risk of suicide (Goldsmith et al. 2002). The term *gatekeepers* also refers to the members of the community, including significant others, peers and co-workers, who are able to act in different ways in order to prevent suicide, e.g. by facilitating referral (Isaac et al. 2009). For example, a study of gatekeeper training for 231 college students by Rallis et al. (2018) suggests that this particular population may hold implications for suicide prevention.

It seems necessary to clarify the extent to which the student population plays a role in gatekeeping. In many countries, the college population, and young adults in general, are considered to have relatively high exposure to suicide (Aldrich 2015; Makaruk et al. 2018; Rotenstein et al. 2016). Students interact regularly with each other and research shows that when facing a crisis, they are more likely to seek support from friends than the mental health services (Drum et al. 2009). Vogel et al. (2007) report examples of students encouraging those at risk to seek professional help, which, in fact, could be regarded as gatekeeping by early intervention.

On graduation, Psychology, Law and Medicine students can be regarded as being part of the way to developing their capability to provide emotional, general health and mental health support, or legal assistance; however, it is not easy to predict how many would have a high level of exposure to suicide at their future workplaces. In general, while university students may be regarded as playing the role of lay gatekeepers, students of the helping professions could be argued to have particular importance in suicide prevention. This subgroup is extensively educated to provide different types of professional support, and many of their graduates may go on to work in professions which are recognized to have substantial or high priority for gatekeeping.

Mental health, ATS and suicidal behavior are known to be interrelated (e.g., Cavanagh et al. 2003; Gibb et al. 2006). Psychological well-being is associated with mental health, and both aspects should be included in any holistic approach towards preventive health and positive functioning. Both factors could influence suicide prevention within both the general population and that of potential gatekeepers, although this phenomenon has been only lightly discussed (Burnette et al. 2015; Hirsch et al. 2018; Lygnugaryte-Griksiene et al. 2017; Quinnett 2018). It has been suggested that the well-being of providers and their attitudes towards the end-of-life decision are important factors in bringing hope and preventing suicide (Burnette et al. 2015; O’Hara 2013). This view is supported by Foulds (1969) and Porter (1995) who consider that the well-being of providers achieved by self-actualization empowers client growth.

Surprisingly, no previous studies have evaluated the prediction of ATS and ATSP based on a comprehensive mental health model incorporating indicators of positive and negative functioning. However, such an approach has been proposed by the dual-factor model of positive mental health (DFM) (Greenspoon and Saklofske 2001), which was originally introduced to address the limitations of traditional health models. The DFM characterizes complete mental health as a combination of high psychological, or subjective, well-being with a low level of psychopathology. Indeed, Joseph and Wood (2010) argue that positive and negative functioning could be seen as interrelated constructs, constituting a higher-order continuum of holistic well-being.

Although there is no consensus regarding the list of indicators that can be used to determine psychopathology and well-being for the DFM, the positive and negative indicators of mental health can be seen as two continua, with the upper end often conceptualized as flourishing, and the lower end defined as languishing (Keyes 2002; Wang et al. 2011). Regarding the indicators that can be included in the positive continuum, Keyes (2005) identifies emotional, psychological and social well-being, which he named the “complete state model of mental health” (Keyes 2005, p. 539). The way the DFM integrates symptoms with personal resources makes it a comprehensive form of assessment. Assessments of personal resources have been empirically confirmed to play an important role in predicting states of impaired functioning, such as depression, even when controlled for negative functioning (Wood and Joseph 2010).

Hence, there is a need to evaluate the potential of DFM with regard to predicting ATS and ATSP, since previous analyses have focused mainly on mental health problems (Eskin et al. 2016; Gibb et al. 2006; Sallander Renberg and Jacobsson 2003). In addition, little attention has been also paid to assessing the role of positive indicators for predicting ATS and ATSP among the potential recipients of gatekeeping training. Furthermore, few studies have examined the relationship between positive mental health and ATS among different groups of gatekeepers, and this may have cast a shadow across many training programs aimed at this population. Most studies demonstrate a narrow focus on increasing knowledge on risk identification or teaching how to develop an intervention plan which could increase self-efficacy, but fail to promote optimal functioning (Isaac et al. 2009).

For the purpose of this study, it is important to distinguish two terms which are applicable to DFM and are related to well-being and suicide theories. Life *meaningfulness* and *meaninglessness* are referred to in diverse psychological concepts within both positive and pathogenic paradigms, including Ryff’s eudaimonic approach to psychological well-being, models of hope, pro-preventive orientation towards suicide vs. Beck’s negative triad, Shneidman’s cognitive constriction or orientation towards suicide as a solution, respectively (Delle Fave et al. 2011; Gibb et al. 2006; Ringel 1983; Ryff 1989; Sallander Renberg and Jacobsson 2003; Shneidman 1985; Snyder et al. 1991; Wenzel and Beck 2007). The interdependence of life *meaningfulness* (related to eudaimonia) and *meaninglessness* (related to suicidality) could be framed in two ways: (a) they are organized within a single continuum ranging from positive to negative functioning, or (b) they form a hierarchically-organized structure, where negative functioning influences well-being, i.e. the highest level of the hierarchy (see: Joseph and Wood 2010). The latter approach suggests that to investigate their relationship with ATS and ATSP, measures of both negative and positive functioning, i.e. those fitting the assumptions of the DFM, should be integrated.

For gatekeepers, finding meaning in life plays an important role in predicting their helping behavior and valuing the end-of life decision. Meaningfulness is an important aspect of eudaimonia, which defines happiness as personal growth, finding meaning in life and maintaining self-actualization (Ryff 1989). Maintaining good eudaimonic well-being among providers ensures that their hope and authenticity may overcome the difficulties presented by a client (Foulds 1969; O’Hara 2013); such progress can be achieved by the client as a result of internalization, behavioral modeling and positive effect of self-disclosure (Knox and Hill 2003; Kottler 2017).

These findings suggest that by focusing on the self-actualization of providers, it is possible to increase positive functioning, which is desirable for communicating emphatic understanding and facilitating genuineness. Facilitating the personal resources of those who intervene with a suicidal or dying person is a particularly important goal, as helping behavior can entail a number of undesirable outcomes such as emotional exhaustion, compassion fatigue and burnout (Benson and Magraith 2005; Marquis 2005). This may contribute to the development of negative attitudes towards those who are likely to die, manifested by avoidance or low collaboration (Nia et al. 2016).

If *life meaningfulness*, represented by eudaimonia, could be at one extreme, *meaninglessness* would be at the other. For example, according to the escape theory of suicide, the experience of meaninglessness could be characterized as a stage in a suicidal crisis, resulting from dissociating from an unhappy state into cognitive deconstruction (Baumeister 1990). Regarding the existential approach towards suicidality, meaninglessness is experienced more consciously as an individual’s choice and a solution to existential dilemmas (Orbach 2007; Rogers 2001). In fact, from an existentialist perspective, meaningfulness could be regarded as the outcome of dealing with the universal fear of death by transcending it into focusing on a meaningful life; this can be achieved, for example, by embracing religious systems (Becker 1997) or engaging in self-actualization attained by fulfilling intrinsic psychological needs (Ryff 1989). Similarly, meaninglessness could arise through the transcendence of fear of death into death fearlessness (Chu et al. 2017; Grewal and Porter 2007), which has implications for ATS and ATSP.

The absence of mental health concerns among providers does not give a comprehensive view on their functioning; such an approach may fail to identify those who are languishing in terms of positive indicators of well-being and are likely to develop mental illness (Suldo and Shaffer 2008). For example, low hopers are more prone to developing suicide risk (Davidson et al. 2009), suggesting that permissive ATS plays a mediating role. Furthermore, providers with low positive functioning are thought to harbor more negative attainable expectations towards themselves and others, and to be less likely to discover and pursue purposeful goals (Snyder et al. 1991) which impedes the establishment of a supportive relationship (Brossart et al. 1998; O’Hara 2013), and possibly lessen the preparedness and willingness of the gatekeeper to prevent suicide. The working alliance with the client is determined by the ability of the provider to formulate realizable goals and tasks, as well as by their beliefs about the expected outcomes. The personal development of the providers affects their ability to see possibilities beyond the present struggle, as well as their belief in the capacity for the client to change and their belief in the power of prevention and intervention (O’Hara 2013; Snyder et al. 1991).

ATS and ATSP reflect the beliefs held by the provider that intervention is manageable and meaningful (Sallander Renberg and Jacobsson 2003), which suggests that hopefulness and *life meaningfulness* serve as related themes to pro-preventive orientation. Pro-preventive orientation is a construct derived from research on ATS and ATSP (Sallander Renberg and Jacobsson 2003), which could be defined as the subjective preparedness and willingness to intervene personally; it also involves positive cognitive expectations towards prevention. Low pro-preventive orientation may therefore indicate individual disengagement from initiating the intervention (Sallander Renberg and Jacobsson 2003; Stecz 2019).

A number of important sources of attitudes towards suicide exist in the literature. They are framed by cultural norms, religious beliefs and the job environment, which differ across societies and particular communities (Gearing and Lizardi 2009; Hjelmeland et al. 2008; Lee et al. 2007; Park et al. 2016; Poreddi et al. 2016; Sisask et al. 2010). Several studies suggest that sociodemographic variables such as male gender, higher economic status or being married were associated with more a pro-preventive orientation towards suicide (Poreddi et al. 2016); however, the effect of gender and marital status vary between countries (Lee et al. 2007; Park et al. 2016). Secondly, ATS is argued to be associated with psychological problems related to general mental health. Psychological distress and decreased subjective well-being have been found to be associated with more accepting ATS but more rejecting approach towards suicidal persons (Eskin et al. 2016).In addition, permissive ATS are thought to be potential risk factors for suicidal behavior and to be related to mental disorders: the prevalence of mental disorders at the time of death among those who commit suicide is considered to exceed 90% (Herrera 2018). Finally, psychological well-being, comprising purpose in life and positive expectations towards life, could be argued to affect the orientation towards suicide prevention.

The clinical implications of the model could be applicable for a wide range of helping situations.

The variability of ATS could be explained by the variation in the aspects of happiness, which are acknowledged to have a complex framework (Delle Fave et al. 2011). Bringing psychological symptoms and psychological well-being together integrates two approaches towards positive mental health, as demonstrated by the DFM. This corresponds well with the debate taking place within positive psychology, which integrates different views on happiness and embraces both the negative and positive aspects of human life (Ivtzan et al. 2015).

Previous research has linked suicidality and permissive ATS with low hedonistic well-being and hopelessness in both general and clinical populations (Cheavens et al. 2016; Davidson et al. 2010; Joiner et al. 2001; Yamokoski et al. 2011): findings which consolidate theories in suicidology and positive psychology in some regard. However, the relationship between eudaimonic well-being and ATS/ATSP seems to be underinvestigated, and our understanding of the relevance of the happiness felt by potential and future gatekeepers toward their effectiveness at suicide prevention has hence evolved in a fragmented way.

### Aim of the Study

Our research question concerns whether it is possible for psychological well-being to predict ATS and ATSP among students of the helping professions, controlling for sociodemographic characteristics, religiousness and psychological problems related to their general mental health. It was hypothesized that higher level of eudaimonic well-being would have predictive validity for stronger orientation towards suicide prevention when controlling for sociodemographic variables (gender, economic situation, marital status), religious beliefs and psychological problems related to mental health.

We argue that the hierarchical view of ATS and ATSP among the students of the helping professions, reflecting future gatekeepers, should be awarded further consideration as a framework. First, ATS and ATSP might be associated with sociodemographic and personal variables (e.g. gender, marital status, economic status, religiousness), as shown in previous research (Bagley and Ramsay 1989; Lee et al. 2007; Park et al. 2016; Poreddi et al. 2016). Secondly, permissive ATS and low orientation towards suicide prevention are expected to demonstrate a relationship with higher levels of emotional distress, somatic complaints, anxiety and depression, comprising decreased general mental health and lower subjective well-being as indicated by General Health Questionnaire (GHQ). Finally, ATS and ATSP would be predicted by eudaimonic happiness, which should serve a preventative function even when risk factors occur. A hierarchical model might demonstrate the effect of psychological well-being on pro-preventive orientation, controlled for confounding variables.

## Method

### Participants

The study was approved by the University of Łódź Bioethics Committee and all participants gave their informed consent to participate. Participation was fully voluntary and was not supported financially nor with the offer of course credits; similarly, those who declined to take part were not placed under any pressure to do so. The sample (N = 239) consisted of students of the helping professions (Law, Medicine, Psychology) from two public universities in Central Poland.

The rationale for considering Law, Medicine and Psychology students as study participants was that gatekeeping involves different agents and employs a wide range of interventions comprising health, psychological and legal support. In addition, a more heterogeneous group was formed in order to increase external validity of the results.

All students enrolled in the obligatory course were invited to take part in the study, with 98.4% agreeing to do so. Women (n = 166) comprised 69.5% of the sample, which moderately affected sample specificity (one sample χ^2^(1) = 36.188, φ = 0.39, *p* < 0.001). The ethnicity of the sample was Caucasian. The demographic characteristics of the participants are outlined in Table 1.

**Table 1:** Sample characteristics

Characteristics	Description	N	%	M	SD	Sex distribution
Age	–	–	–	22.84	5.15	n.s.
Marital status	Single	109	45.6			More single males χ^2^(3) = 12.487 *p* = .006
	Coupled or married	121	50.6			
	Divorced or separated	7	2.9			
	Unknown	2	0.9			
Economic status	Low	3	1.3			n.s.
	Medium	127	53.1			
	High	109	45.6			
Residential area	Rural	43	18.0			n.s.
	Semi-urban	27	11.3			
	Urban (cities above 25,000 citizens)	169	70.7			

n.s.—not significant

### Instruments

#### Psychological Wellbeing Scale (PWB-42)

The Psychological Wellbeing Scale, developed by Ryff (1989) and adapted in Poland by Krok (2009) is a statistically-sound and theoretically-driven questionnaire based on the eudaimonic concept of happiness. Respondents use a seven-point Likert scale to rate how much they agree or disagree with 42 statements measuring six aspects of well-being and happiness: self-acceptance, purpose in life, positive relations with others, personal growth, environmental mastery and autonomy (i.e. “I have confidence in my opinions, even if they are contrary to the general consensus”). Cronbach’s internal consistency coefficients for psychological well-being subscales range from α = .68 (personal growth) to α = .85 (self-acceptance), with values of α = .69 (purpose in life), α = .77 (positive relations with others), α = .81 (autonomy) and α = .84 (environmental mastery) observed in our sample.

#### Questionnaire on Attitudes Towards Suicide (ATTS)

The ATTS developed by Sallander Renberg and Jacobsson (2003) is a self-report scale used to assess ATS and ATSP. The Polish ATTS version with 18 items has been demonstrated to have a unique five-factor structure, with McDonald’s Omega coefficients varying from .57 (*suicide preventability*—*cognitive*) to .85 (*suicide as a right*) and has shown generally adequate reliability and acceptable construct validity (Stecz 2019). In our sample, most internal consistency reliabilities were acceptable, with *suicide as a right (Cronbach’s α *=* .84), suicide as a solution (α *= *.68), suicide (in)comprehensibility (α *= *.66), suicide preventability*—*cognitive (α *= *.55)* and *orientation towards suicide prevention (α *= *.60)*. Scores range from 3 to 18, except for the six-item subscale *suicide as a right*, which ranges from 6 to 30. We analyzed four domains (*suicide preventability*—*cognitive, orientation towards suicide prevention, suicide (in)comprehensibility, suicide as a solution*) which were considered to be the most central for the purpose of this study.

#### General Health Questionnaire (GHQ-28)

The GHQ-28 developed by Goldberg (1978) is intended for use as a self-report screening instrument for detecting the possibility of psychological disorder. The GHQ-28 consists of 28 statements divided into four subscales: *somatic symptoms* (hypochondria), *anxiety and insomnia*, *social dysfunction* (understood as inability to pursue activities), and *severe depression* (e.g., *“found yourself wishing you were dead and away from it all”*). The overall score comprising 28 items ranges from 0 to 84. High scores suggest emotional distress and low subjective well-being associated with risk of general and mental health problems. The GHQ-28 contains several positively-worded items (e.g. “have been feeling reasonably happy”) with the response option “more so than usual” so that lower scores could be interpreted as higher level of functioning in positive categories (Joseph and Wood 2010), providing a measure of subjective (emotional) well-being. The Polish validation demonstrated adequate psychometric properties and reproduced its factorability (Goldberg et al. 2001); similarly, the GHQ-28 demonstrated acceptable reliability in our sample (α = .93), with satisfactory internal consistency values for the somatic symptoms (α = .74), anxiety (α = .86), dysfunction (α = .85), and depression (α = .90) subscales. Although all GHQ-28 subscales represent operational variables related to psychological and mental health problems, they are closely correlated with each other (r ranged from .40 to .66; p < .001); therefore, the general GHQ-28 score was used in the analyses in the present study to provide a more holistic approach.

#### Centrality of Religiosity Scale (CRS)

The scale was developed by Huber (Huber 2003; Huber and Huber 2012) for measuring the salience of religious meanings in the individual. The five core dimensions, considered to be representative for the total of religious life, were: *worship* (public practice), *religious or spiritual experience*, *prayer*, *religious beliefs* and *religious interests*. The instrument also allows the measurement of the general importance of religion for the individual. Polish version, provided with 15 items, demonstrated adequate psychometric properties (Zarzycka 2007), as reported in our sample by Cronbach’s alpha for *overall centrality of religiosity* (α = .95). General religiousness score range from 15 to 75 with higher scores reflecting higher levels of religious centrality in the individual’s life.

### Statistical Analyses

Preliminary analyses, independent samples t-tests, Spearman and Kendall’s rank correlations, Pearson correlations (when the assumptions were not violated) and three-step hierarchical regression analyses were conducted. Robust 95% CIs were computed for all correlation and regression coefficients using bootstrapping with 1000 samples. Bonferroni’s correction was applied for familywise Type I error in correlation analysis. All calculations were performed using IBM SPSS Statistics 24.

### Preliminary Analysis—Outlier Detection

Prior to conducting the analysis, a sample size of 239 subjects was considered to be adequate, given the originally planned 12 independent variables (including demographic variables, religiousness, general mental health problems and dimensions of PWB) used in the hierarchical multiple regression (Tabachnick and Fidell 2013). Data were tested for violations in distribution and the presence of multivariate outliers for testing the relevant assumptions of multiple regression. None of the absolute values of skewness (all above − .89) or kurtosis (all above − 1.54) indicated substantial violations of normality (e.g., Kline 2016). No extreme multivariate outliers were detected in data sets for prediction of ATS/ATSP.

## Results

No significant differences were found in ATS and ATSP with regard to gender or marital status (Tables 2 and 3).

**Table 2:** Independent samples *t* tests according to gender (*N* = 239)

Sex	N	Mean	SD	SE mean	t	df	p*
*Suicide as a solution*							
Women	166	11.54	2.40	.19	− .36	237	.72
Men	73	11.66	2.41	.28			
*Preventability*—*cognitive*							
Women	166	6.50	2.87	.22	− .82	237	.43
Men	73	6.84	3.00	.35			
*Suicide (in)comprehensibility*							
Women	166	8.58	2.80	.22	.61	237	.54
Men	73	8.34	2.89	.34			
*Orientation towards suicide prevention*							
Women	166	12.20	2.23	.173	1.59	237	.12
Men	73	11.68	2.44	.29			

*Significance testing based on bootstrapped SE. Bootstrap based on 1000 samples

**Table 3:** Independent samples *t* tests according marital status (*N* = 230)

Marital status	N	Mean	SD	SE mean	t	df	p*
*Suicide as a solution*							
Single	109	11.73	2.36	.23	.82	228	.41
In relationship	121	11.47	2.47	.22			
*Preventability*—*cognitive*							
Single	109	6.59	3.03	.29	.11	228	.91
In relationship	121	6.55	2.82	.256			
*Suicide (in)comprehensibility*							
Single	109	8.56	2.97	.28	.28	228	.78
In relationship	121	8.45	2.68	.24			
*Orientation towards suicide prevention*							
Single	109	12.09	2.37	.23	.22	228	.83
In relationship	121	12.02	2.23	.20			

*Significance testing based on bootstrapped SE. Bootstrap based on 1000 samples. Data for marital status were missing for nine participants

No significant relationships were found between ATS/ATSP and socioeconomic status (τ_b_ = − .04 with *suicide as a solution*; τ_b_ = − .08 with *preventability*—*cognitive*; τ_b_ = .02 with *suicide (in)comprehensibility* and τ_b_ = .04 with *orientation towards suicide prevention*; *p* > .05) and age (r_s_ = .06 with *suicide as a solution*; r_s_ = .01 with *preventability*—*cognitive*; r_s_ = − .11 with *suicide (in)comprehensibility* and r_s_ = − .05 with *orientation towards suicide prevention*; *p* > .05). Therefore, these variables were not included in further analyses.

Descriptive statistics for ATS/ATSP, religiousness, general mental health problems and dimensions of psychological well-being are shown in Table 4, together with correlations between the variables. Significant correlations were found between *suicide (in)comprehensibility* and religiousness; *preventability*—*cognitive/suicide (in)comprehensibility* and general health problems. Regarding dimensions of psychological well-being, significant correlations were observed between all dimensions and *preventability*—*cognitive*; *suicide (in)comprehensibility* correlated with environmental mastery and self-acceptance; and *orientation towards suicide prevention* correlated with positive relations with others and purpose in life. On the other hand, *suicide as a solution* did not show any significant correlation with dimensions of psychological well-being (Table 4).

**Table 4:** Descriptive analysis and correlation analysis (*N* = 239)

	1	2	3	4	5	6	7	8	9	10	11	12
Mean	11.57	6.60	8.51	12.04	40.14	26.69	34.37	32.56	36.25	37.08	36.71	33.00
SD	2.40	2.91	2.83	2.31	15.57	13.51	7.62	8.09	6.02	7.03	6.62	8.00
1. Suicide as a solution	1											
2. Preventability—cognitive	.157	1										
3. (In)comprehensibility	− .102	.505**	1									
4. Orientation towards suicide prevention	.397**	− .183*	− .176	1								
5. Religiousness	.122	− .149	− .310**	.145	1							
6. General mental health problems	− .096	.392**	.264**	− .029	− .056	1						
7. Autonomy	.122	− .254**	− .110	.039	− .170	− .317**	1					
8. Environmental mastery	.137	− .459**	− .220**	.089	.005	− .571**	.517**	1				
9. Personal growth	.123	− .315**	− .105	.173	.067	− .310**	.486**	.635**	1			
10. Positive relations with others	.136	− .266**	− .145	.252**	.195*	− .247*	.141	.522**	.467**	1		
11. Purpose in life	.146	− .305**	− .098	.197*	.094	− .319**	.321**	.528**	.573**	.387**	1	
12. Self-acceptance	.169	− .437**	− .181*	.091	− .059	− .558**	.589**	.778**	.673**	.458**	.559**	1

*p < .05 after applying Bonferroni correction for familywise Type I error

**p < .01 after applying Bonferroni correction for familywise Type I error

Four sets of hierarchical multiple regression analyses were conducted to determine the role of psychological well-being in predicting ATS and ATSP. Each regression analysis included religiousness (stage 1), general mental health problems (stage 2) and six dimensions of psychological well-being (stage 3). The results of the multiple regression analyses are given in Tables 5, 6, 7 and 8.

**Table 5:** Results of multiple regression analysis (dependent variable: *suicide as a solution*)

Step	Predictors (*N *= 239)	*B*	95% CI of B*		*SE*_B_^*^	β	p*
			Lower	Upper			
1	Religiousness	.019	− .002	.038	.010	.122	.057
2	Religiousness	.018	− .002	.037	.010	.117	.076
	General mental health problems	− .016	− .043	.007	.013	− .090	.236
3	Religiousness	.020	− .001	.042	.011	.131	.065
	General mental health problems	.002	− .025	.030	.014	.011	.883
	Autonomy	.027	− .030	− .087	.030	.085	.379
	Environmental mastery	− .010	− .086	.071	.041	− .035	.814
	Personal growth	− .023	− .063	.101	.042	− .057	.572
	Positive relations with others	.020	− .105	.063	.027	.060	.464
	Purpose in life	.022	− .041	.084	.032	.061	.471
	Self-acceptance	.041	− .065	.135	.051	.136	.422

*Significance testing based on bootstrapped SE. Bootstrap based on 1000 samples

Step 1: R^2^ = .015, F(1, 237) = 3.560, *p* > .05

Step 2: R^2^ = .023, F(2, 236) = 2.754, *p* > .05, ΔR^2^ = .008, F(1, 236) = 1.935, *p* > .05

Step 3: R^2^ = .054, F(8, 230) = 1.635, *p* > .05, ΔR^2^ = .031, F(6, 230) = 1.256, *p* > .05

**Table 6:** Results of multiple regression analysis (dependent variable: *preventability*-*cognitive*)

Step	Predictors (*N *= 239)	*B*	95% CI of B*		*SE*_B_^*^	β	p*
			Lower	Upper			
1	Religiousness	− .028	− .052	− .003	.012	− .149	.020
2	Religiousness	− .024	− .047	− .001	.012	− .128	.037
	General mental health problems	.083	.050	.118	.017	.385	.001
3	Religiousness	− .029	− .051	− .007	.011	− .153	.016
	General mental health problems	.032	− .003	.075	.019	.146	.091
	Autonomy	− .005	− .061	.050	.028	− .013	.851
	Environmental mastery	− .083	− .156	− .012	.037	− .232	.027
	Personal growth	.018	− .063	.101	.041	.036	.629
	Positive relations with others	.002	− .059	.058	.030	.004	.957
	Purpose in life	− .017	− .090	.054	.037	− .039	.631
	Self-acceptance	− .066	− .138	.011	.038	− .180	.076

*Significance testing based on bootstrapped SE. Bootstrap based on 1000 samples

Step 1: R^2^ = .022, F(1, 237) = 5.417, *p* = .021

Step 2: R^2^ = .170, F(2, 236) = 24.201, *p* < .001, ΔR^2^ = .148, F(1, 236) = 42.047, *p* < .001

Step 3: R^2^ = .268, F(8, 230) = 10.526, *p* < .001, ΔR^2^ = .098, F(6, 230) = 5.122, *p* < .001

**Table 7:** Results of multiple regression analysis (dependent variable: *suicide (in)comprehensibility*)

Step	Predictors (*N *= 239)	*B*	95% CI of B*		*SE*_B_^*^	β	p*
			Lower	Upper			
1	Religiousness	− .056	− .076	− .034	.011	− .310	.001
2	Religiousness	− .054	− .073	− .033	.010	− .296	.001
	General mental health problems	.052	.029	.076	.012	.248	.001
3	Religiousness	− .060	− .081	− .037	.011	− .328	.001
	General mental health problems	.034	.004	.065	.016	.161	.029
	Autonomy	− .028	− .086	.028	.029	− .076	.335
	Environmental mastery	− .045	− .115	.027	.037	− .128	.215
	Personal growth	.045	− .031	.134	.042	.096	.267
	Positive relations with others	− .001	− .074	.065	.035	− .002	.991
	Purpose in life	.024	− .037	.088	.032	.057	.429
	Self-acceptance	− .022	− .100	.055	.039	− .062	.567

*Significance testing based on bootstrapped SE. Bootstrap based on 1000 samples

Step 1: R^2^ = .096, F(1, 237) = 25.178, *p* < .001

Step 2: R^2^ = .157, F(2, 236) = 22.000, *p* < .001, ΔR^2^ = .061, F(1, 236) = 17.111, *p* < .001

Step 3: R^2^ = .177, F(8, 230) = 6.170, *p* < .001, ΔR^2^ = .020, F(6, 230) = .910, *p* > .05

**Table 8:** Results of multiple regression analysis (dependent variable: *orientation towards suicide prevention*)

Step	Predictors (*N *= 239)	*B*	95% CI of B*		*SE*_B_^*^	Β	p*
			Lower	Upper			
1	Religiousness	.021	.001	.040	.010	.145	.030
2	Religiousness	.021	.001	.040	.010	.144	.032
	General mental health problems	− .004	− .026	.016	.011	− .021	.734
3	Religiousness	.012	− .007	.031	.010	.083	.223
	General mental health problems	.001	− .021	.024	.012	.007	.917
	Autonomy	.013	− .030	.063	.023	.042	.579
	Environmental mastery	− .036	− .100	.029	.032	− .126	.259
	Personal growth	.033	− .039	.110	.037	.085	.386
	Positive relations with others	.077	.027	.133	.027	.235	.003
	Purpose in life	.051	.008	.101	.024	.145	.039
	Self-acceptance	− .021	− .088	.037	.031	− .074	.498

*Significance testing based on bootstrapped SE. Bootstrap based on 1000 samples

Step 1: R^2^ = .021, F(1, 237) = 5.4078, *p* = .025

Step 2: R^2^ = .021, F(2, 236) = 2.584, *p* = .078, ΔR^2^ < .001, F(1, 236) = .109, *p* > .05

Step 3: R^2^ = .098, F(8, 230) = 3.139, *p* < .01, ΔR^2^ = .077, F(6, 230) = 3.274, *p* < .01

No significant predictor was found for *suicide as a solution*. Moreover, whole models 1 to 3 were also not significant (Table 5).

As seen in Table 6, religiousness and general mental health problems significantly predicted the *preventability*—*cognitive* attitude towards suicide (stage one and two). Religiousness remained significant even after adding general mental health problems (step two) and psychological well-being dimensions (stage three). The only significant predictor from psychological well-being dimensions was environmental mastery. Psychological well-being accounted for additional 9.8% of the variance in *suicide preventability*—*cognitive* and was the most important predictor in the regression model.

Religiousness and general mental health problems significantly predicted *suicide (in)comprehensibility* (Table 7). Both variables remained significant even after adding psychological well-being dimensions into the regression model (stage two and three). In contrast, psychological well-being was not a significant predictor of *suicide (in)comprehensibility* when introduced at stage three. The significant increase of explained variance in the model was observed only at stage two after adding general mental health problems (stage two).

*Orientation towards suicide prevention* (Table 8) was significantly predicted by religiousness (stage one) and two dimensions of well-being (introduced at stage three): positive relations with others and purpose in life. Religiousness remained significant when controlled for general mental health problems (stage two), but was not significant when all independent variables were included in stage three. Contrary to the previously-examined ATSP, the presence of general mental health problems was not a significant predictor. Moreover, the addition of psychological well-being variables to the regression model at stage three explained an additional 8% of the variation in *orientation towards suicide prevention*, and this change was significant.

## Discussion

The role of gatekeepers is not restricted to identifying suicide risk or making referrals to crisis intervention services. From a positive psychology perspective, providers, also known as designated gatekeepers, serve as significant others to those in crisis, and thus may facilitate personal growth and rehabilitation of those at suicide risk. Authenticity and positive regard towards a person experiencing *life meaninglessness* are thought to work effectively if they correspond with provider’s own self-actualization and positive functioning (Foulds 1969; O’Hara 2013). The central assumption of the present study was that the positive mental health of future gatekeepers could be related to a pro-preventive orientation towards suicide, which would have consequences for social policy.

The study was directed towards students of the helping professions. These participants are future gatekeepers, and have been found to score lower on eudaimonic well-being, controlled for religiousness and symptoms of psychological disorders; as hypothesized, they were found to be oriented less strongly towards suicide prevention. Also, as hypothesized, low eudaimonic well-being predicted more acceptable ATS; however, this result was not significant when controlled for confounding variables. It is somewhat surprising that neither age, gender nor economic status were associated with ATS and ATSP: other findings suggest that cultural differences or sociodemographic variables may influence ATS (Lee et al. 2007; Poreddi et al. 2016; Sallander Renberg and Jacobsson 2003). Although this result may appear confusing, religion is known to play an important role within the Polish community (Storm 2017). Our present findings indicate that religiousness may have a stronger influence on ATS variability than other demographic characteristics (*cf.* Zou et al. 2016). Due to the structure of the sample, the student population does not provide enough generalizability for making unbiased inferences related to demographics.

Our findings suggest that positive relations with others may be a significant predictor of orientation towards suicide prevention. An orientation towards prevention involves an instrumental component, defined as readiness to prevent suicide when approaching a person in crisis (Sallander Renberg and Jacobsson 2003; Stecz 2019). The achievement of close relationships with others then could be seen as the ability to empathize and care. Our findings can be better understood when viewed from the perspective of Ryff’s (1989) arguments linking positive relations with others with the concepts of maturity and generativity, described as providing care for others and feeling warmth for human beings. According to the multidimensional model of psychological well-being, a decreased quality of relationships could be attributed to difficulties with being concerned about others (Ryff 2013); thus, individuals with unsatisfying relationships are less likely to become involved in suicide prevention, which could be explained by high demands for empathy, care and positive regard in the helping professions.

Considering the multidimensionality of psychological well-being and its relevance to suicide theories, it is interesting to note that our results confirm that *purpose in life* is only significantly related to *orientation towards suicide prevention* when controlled by other variables in the proposed model. Other attitude dimensions (*suicide preventability*-*cognitive, suicide as a solution and suicide (in)comprehensibility*) were not found to be related to purpose in life when controlled for confounders. At the same time, this study demonstrated that orientation towards prevention is predicted by *environmental mastery*. This result extends previous studies and deserves further discussion. To clarify the role of *purpose in life* for understanding the formation of schemata related to suicide and suicide intervention, it is necessary to more precisely define *life meaningfulness*. Meaning-making should be then considered as a broader construct and a process referring to human beliefs, subjective feelings and goals, comprised by different subconstructs (Park 2010). Ryff and Keyes (1995) proposes that *purpose in life* is influenced by at least two aspects: the realization of one’s goals and the belief that one’s life is meaningful. It is possible that only some of the subcomponents of *purpose in life* are relevant to ATS in general and to pro-preventive orientation. Furthermore, situational level of *purpose in life* varies according to stressful events among providers, suggesting that reduced purpose in life could be interpreted as the initiation of change or restoration of meaning-making processes (Skaggs and Barron 2006).

Our findings provide evidence for the generalizability of the DFM, comprising both symptoms of psychological disorders (also interpreted as decreased subjective well-being) and eudaimonic well-being, for studying the predictors of ATS among future gatekeepers, i.e. students of the helping professions. A pro-preventive orientation towards suicide is better explained by a joint model than by any fragmentary model emphasizing either psychological well-being or psychological complaints. Current model is also controlled for centrality of religiousness, which is known to be of high importance in predicting ATS. A study based on a Turkish sample found religiousness to be associated with stigmatizing ATS (Vatan et al. 2010). In a sample on Christian adults, Domino and Miller (1992) report greater religiousness to be related to perceiving suicide as a unforgivable moral evil. Our study revealed that religious beliefs and faith predicted suicide incomprehensibility, which highlights the importance of negative emotions and evaluations associated with suicide perception among more religious potential gatekeepers and confirms that our results are consistent with those of earlier studies.

Although our hypotheses were supported, the relationship between psychological well-being domains and ATS/ATSP is not as strong as expected. The findings might indicate that the constructs of well-being and pro-preventive orientation are linked by different mechanisms than assumed. A possible explanation for identifying *environmental mastery* as a significant predictor of pro-preventive orientation could be that helping behavior and the preparedness to help in difficult situations have been demonstrated to be dependent on specific self-evaluations, such as self-efficacy (Bandura 1995; Jerusalem and Schwarzer 1992; Lipson 2014). *Environmental mastery* has been defined by Ryff (1989) as a sense of competence in managing activities in accordance with personal needs and values. Negative expectations about managing and improving the surrounding context refer to pessimistic appraisals and predict negative interaction regulation if self-efficacy is deficient (Knopp and Delle-Fave 2013). The underlying causes for negative expectations and pessimistic attitudes have been described extensively in literature through the lenses of a fixed *versus* open mindset (Dweck 2006), attribution hypothesis (Abramson et al. 1978), expectation bias (Coats et al. 1996; Rosenthal and Jacobson 1992) or nocebo effect (Corsi and Colloca 2017).

Due to certain limitations of the study design, these findings cannot be extrapolated to all groups of the gatekeepers and need to be interpreted with caution. The cross-sectional design and convenient sampling method preclude a definitive discussion of the causal relationships between well-being and pro-preventive orientation. In addition, the sample consisted of undergraduate students, who play a role in gatekeeping but have limited experience in prevention, and data collection was performed during their educational training, before establishing professional career. Our results, could not be therefore generalized on the population of actual gatekeepers. Our attempt to recruit a wide range of potential future gatekeepers (including Law students) could be also regarded as a trade-off between external and internal validity. Finally, some caution should be observed when interpreting the results, as the internal consistencies of the ATTS subscales were a little lower; in addition, the available questionnaires on ATS and ATSP rely on self-report, and as they generally have rather limited psychometric characteristics, they can be exposed to measurement bias (Kodaka et al. 2011; Stecz 2019).

Furthermore, the data demonstrated significant results, but the described models did not account for a large proportion of the variance observed in ATS/ATSP. Hence, there is abundant room for conceptualizing a more complex theoretical model related to DFM for predicting ATS and ATSP. For example, helping behavior has been previously explained within the context of hope theory (O’Hara 2013), compassion and self-compassion effect on increased effectiveness (Raab 2014) or by self-efficacy theory, considering gatekeeper training programs focused on knowledge and skills improvement (Lipson 2014). A number of other variables, including cultural norms (Range et al. 1999), value system (Gagnon and Hasking 2012) or previous exposure to suicidal behavior (see: Batterham et al. 2013) could also be associated with ATS. Finally, our assumptions about well-being, emphasizing the importance of eudaimonia for transforming fear of death into the orientation towards life meaningfulness may leave hedonistic adaptation unnoticed. Further research should rather focus on determining and comparing an alternative model of positive mental health for predicting pro-preventive orientation, which integrates eudaimonic with hedonistic happiness.

Generally, the present work seeks to improve the understanding of the role played by the positive functioning of the potential future gatekeepers in developing a helping orientation towards a person in crisis. In this regard, our study has significant meaning for integrating theory and delivering practical implications for suicide prevention.

## Electronic supplementary material

Below is the link to the electronic supplementary material.

Supplementary material 1 (XLSX 227 kb)
